# Glucocorticoids save lives in COVID-19 patients

**DOI:** 10.7150/ijbs.49125

**Published:** 2020-07-07

**Authors:** Chu-Xia Deng

**Affiliations:** Editor in Chief, International Journal of Biological Sciences; Chair Professor, Faculty of Health Sciences, University of Macau, Macau SAR, China

**Keywords:** Glucocorticoid, COVID-19

## Abstract

Recent studies showed that glucocorticoid drugs, which are easily available as pills on pharmacy shelves worldwide, could save lives of COVID-19 patients. With the swiftly increasing infections of the SARS-CoV-2 pandemic at a lethality rate of about 4.7% countless lives may be saved globally.

The Coronavirus Disease-19 (COVID-19) is caused by the Severe Acute Respiratory Syndrome Coronavirus 2 (SARS-CoV-2). Compared with its close related coronavirus family member, SARS-CoV and MERS-CoV, which infected 8096 people in year 2003 and 2494 people in year 2012, respectively, the SARS-CoV-2 is much more infectious and widespread 1, 2. Started in late 2019, COVID-19 pandemic has spread to over 213 countries and territories around the world, infected more than 11.3 million people and caused over 530,000 deaths already.

Despite great efforts in combating this virulent and deadly disease, no effective treatment regimen has been identified yet. In this issue, Xiang et al. focused on the molecular target angiotensin-converting enzyme 2 (ACE2) of SARS-CoV-2 and conducted a bioinformatics-based screening for ACE2 agonists by differentially expressed genes between ACE2^high^ and ACE2^low^groups. Their comparative study identified several glucocorticoids as ACE2 activators, with hydrocortisone that showed the strongest effect on ACE2 activation and inhibition of IL-6 production, followed by prednisolone, dexamethasone, methylprednisolone, and triamcinolone (Figure 1). After retrospectively analyzing the therapeutic efficacy of nine severe or critical patients from a cohort of 90 COVID-19 cases, who received medium to small doses of glucocorticoids, they found seven out of nine patients displayed significant improvements in clinical parameters. These observations provide experimental and clinical evidence that medium-to-low-dose glucocorticoids may play a protective role in the respiratory and digestive systems by activating ACE2 and suppressing cytokine storm. This work, entitled “Glucocorticoids improve severe or critical COVID-19 by activating ACE2 and reducing IL-6 levels” is now published in the current issue of IJBS 3.

Glucocorticoid are steroids that occur naturally in our body, and are known for their effectiveness at reducing inflammation and suppressing the immune system 4. It is believed that one of the causes for multi-organ failure and lethality of COVID-19 patients is cytokine storm, which occurs when an immune system, is overactivated by virus infection 5, 6. Therefore, how to inhibit the cytokine storm has become a focus for the treatment of COVID-19 patients at severe or critical stages. IL-6 is one of the important cytokines released by inflammatory cells, especially macrophage, and is increased in COVID-19 patients at severe-to-critical stages. Therefore, the level of IL-6 is most frequently measured during the period of hospitalization, especially in severe cases of COVID-19 patients by rescuing medical team 5, 6. The inhibition of glucocorticoids to production of IL-6 certainly serves as a good predictive value for suppressing cytokine storm for COVID-19 patients at severe-to-critical stages.

Why should the activation of ACE2 benefits COVID-19 patients? SARS-CoV-2 uses ACE2 as the receptor to infect human cells during early stage of the disease 7. The virus spike protein (S protein) binds to the ACE2 of cells, and largely depletes this specific receptor of host cells 7, 8. However, ACE2 does not simply serve as a receptor for SARS-CoV-2, it is the crucial enzyme for providing protective role in maintaining functions of respiratory system, cardiovascular system, kidneys, and other important organs 7, 9. The infection of SARS-CoV-2 induces a reduction of ACE2 levels and disrupts its protective function for host. Therefore, the repletion of ACE2 might be responsible for the rapidly deterioration to severe or critical conditions of COVID-19 patients. Based on this consideration, Xiang et al. believed that up-regulation of ACE2 by glucocorticoids in the patients with severe or critical condition may also serve as a life-saving mechanism in addition to their role in suppressing cytokine storm 3.

Glucocorticoid drugs, such as hydrocortisone, and dexamethasone can be easily found on pharmacy shelves worldwide and are available as pills. It is a very important finding that glucocorticoid drugs could save lives of COVID-19 patients, as they are easily available and have low costs. Very recently, in a large clinical trial in the United Kingdom, involving about 2,100 participants who received dexamethasone at a low-to-moderate dose of 6 milligrams per day for 10 days, and about 4,300 people who received standard care for SARS-CoV-2 infection, dexamethasone was shown to cut deaths by about one-third in patients who were on ventilators because of coronavirus infection 10. With the current lethality rate of COVID-19 of about 4.7%, countless lives may be saved globally if glucocorticoid drugs continuously stand for their high efficacy for COVID-19 patients.

## Figures and Tables

**Figure 1:**
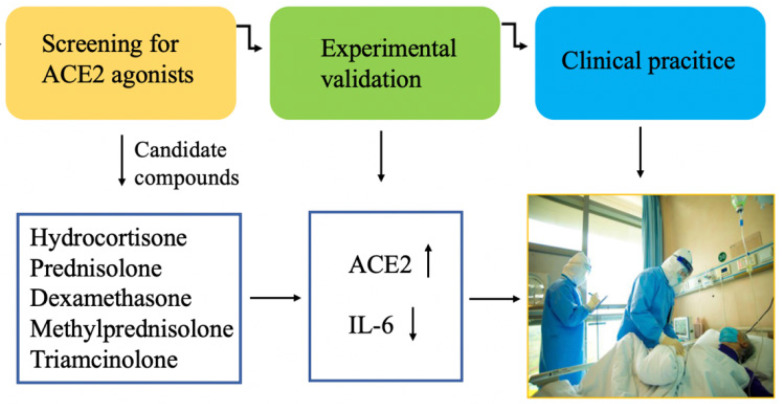
Identification of glucocorticoids as effective drugs for COVID-19 patients. Through bioinformatics-based screening for ACE2 agonists, a group of glucocorticoids were found to increase ACE2 expression and inhibit IL-6 production. Treatment of severe or critical COVID-19 patients revealed significant improvement in clinical parameters. Modified from figures provided by Dr. Yingyan Yu.
